# Association of ANCA associated vasculitis and rheumatoid arthritis: a lesser recognized overlap syndrome

**DOI:** 10.1186/s40064-015-0835-8

**Published:** 2015-02-01

**Authors:** Juliana Draibe, Alan D Salama

**Affiliations:** Nephrology Department, Bellvitge University Hospital, Feixa llarga s/n 08097, Barcelona, Spain; UCL Centre for Nephrology, Royal Free Hospital, London, NW3 2PF UK

**Keywords:** Rheumatoid arthritis, ANCA associated vasculitis, Overlap syndromes

## Abstract

**Background:**

ANCA associated vasculitis (AAV) is an autoimmune disease with significant morbidity and mortality, in which diagnostic delay is associated with worse outcomes. AAV is rarely found in association with other immune mediated diseases. Early recognition of such overlaps enables more timely diagnosis and may impact on disease outcome. We reviewed cases of AAV in which there was an overlap with rheumatoid arthritis (RA).

**Methods:**

We performed a retrospective analysis of our vasculitis database for patients who had a diagnosis of AAV and RA, and a literature search to find other reported cases of this overlap syndrome.

**Results:**

We found six subjects who had a diagnosis of RA and developed AAV at a median of 10.5 years (range 4–43 years) after the diagnosis of RA. They had been treated with a mean of 2 disease modifying drugs (0–4) and all had evidence of renal involvement with median creatinine of 227 μmol/l (range 128–700 μmol/l). Only one had a diagnosis of granulomatosis with polyangiitis, while the rest had a clinical diagnosis of microscopic polyangiitis. Half of the patients had positive rheumatoid factor (RhF) at the time of vasculitis diagnosis, three had MPO-ANCA, one PR3-ANCA, and two had ANCA-negative pauci-immune vasculitis. Additionally, we found 29 other cases reported of this overlap, which also most frequently presented with vasculitic renal manifestations, and were frequently RhF positive at the time of AAV diagnosis.

**Conclusions:**

AAV occurs in subjects with RA rarely, and often with significant delay from the first rheumatological manifestations. Renal involvement is common

## Introduction

Anti-neutrophil cytoplasm antibody (ANCA) associated Vasculitis (AAV) comprises 3 different clinical entities: granulomatosis with polyangiitis(GPA, formerly Wegener’s granulomatosis ), microscopic polyangiitis (MPA) and eosinophilic granulomatosis with polyangiitis (EGPA, formerly Churg-Strauss syndrome ). These clinical syndromes are frequently (but not always) characterized by ANCA reactive against one of two antigens, proteinase-3 (PR3) or myeloperoxidase (MPO), the former more commonly associated with GPA and the latter more frequently found in patients with MPA and EGPA (Finkielman et al. 2007). The genetic basis of these clinical and immunological AAV subsets have recently been described through genome wide association studies and appear to be different between PR3-and MPO-AAV, with the most significant associations with HLA genes, implicated in other autoimmune diseases. (Lyons et al. 2012) Clinically, AAV may rarely be associated with other immune mediated diseases, those that have been well recognized include anti-glomerular basement membrane (GBM) disease (Weber et al. 1992; Kalluri et al. 1997), scleroderma (Derrett-Smith et al. 2013), systemic lupus erythematosus(Tetikkurt et al. 2012) and membranous glomerulonephritis(Gaber et al. 1993; Tse et al. 1997). Knowledge of these overlap syndromes is important in early recognition of potential complications and differences in clinical courses and management pathways.

We have recently noticed development of AAV in a group of patients with Rheumatoid arthritis (RA), where there has been a delay in presentation with vasculitic symptoms following the diagnosis of RA. Here we describe our cohort of patients presenting over the last 10 years and review the literature describing this less well known association. We suggest that recognition of this overlap is important for more effective serological screening and early recognition of potential clinical complications.

### Case 1

A 59 year old male patient was diagnosed with seronegative RA and received treatment with methotrexate (MTX) and prednisolone. Four years after the diagnosis of RA, he presented with a productive cough, haematuria, proteinuria (6.84 g/24 hours) and decline in renal function (serum creatinine (SCr) rising from 119 to 270 mmol/L). He had an acute phase response (CRP 33 mg/L, ESR 134 mm/h) and was PR3-ANCA positive (67 U/ml, normal range 0–3). The RhF and anti-CCP remained negative. A CT scan of the chest showed multiple air filled cavitating structures and CT scan of his sinuses revealed extensive bony defects in the antronasal regions and thickening of the maxillary and frontal sinuses. A kidney biopsy demonstrated focal necrotising vasculitis lesions. As a result a diagnosis of Granulomatosis with polyangiitis(GPA) was made and he was treated with pulsed methylprednisolone, oral prednisolone and intravenous cyclophosphamide. His renal function improved (SCr 115, proteinuria 0.58 g/24 hours) and his pulmonary lesions regressed.

### Case 2

A 54 year old woman diagnosed with seropositive RA after a symmetrical polyarthritis of the metacarpophalangeal joints, wrists and knees developed. She received treatment with infliximab and subsequently MTX and sulphasalazine because of poor disease control. Fourteen years later, she developed acute renal failure (SCr 243 μmol/L) with haematuria and proteinuria (4.58 g/24 hours), elevated acute inflammatory markers (CRP 113 mg/L, ESR 124 mm/h) and anti-MPO positivity (>100 U/ml, NR <10). RhF was highly positive (214 IU/ml, normal range 0–20) as was anti-CCP. Kidney biopsy revealed focal and segmental necrotising glomerulonephritis, with moderate chronic damage. A diagnosis of microscopic polyangiitis(MPA) was made. She was treated with methylprednisolone, oral prednisolone and intravenous pulsed cyclophosphamide and had an improvement in her renal function (SCr 198 μmol/L), proteinuria (0.5 g/24 hours), and CRP (8 mg/L).

### Case 3

A 33 year old woman was diagnosed with seronegative RA. She was intolerant of methotrexate, sulphasalazine and azathioprine, and was treated with etanercept with improvement in her symptoms. Thirty four years later, she developed a vasculitic rash on her legs, and was noted to have acute kidney injury (SCr 211 μmol/L), haematuria and proteinuria (1.55 g/24 hours). She was ANCA negative, but a renal biopsy demonstrated pauci-immune focal necrotizing glomerulonephritis with moderate chronic damage. She was diagnosed with MPA and treated with pulsed methylprednisolone, oral prednisolone and pulsed intravenous cyclophosphamide, following which she had an improvement of renal function (SCr 129 μmol/L) and proteinuria (0.3 g/24 hours).

### Case 4

A 24 year old woman was diagnosed with seronegative RA for which she had therapy with prednisolone. Forty-three years later she was referred to our hospital with declining renal function (SCr 128 mmol/L), associated with proteinuria. Her serum inflammatory markers were elevated (CRP 36 mg/L, ESR 57 mm/h) and MPO-ANCA was positive (124 U/ml), as was her RhF (64 IU/ml, normal range 0–20). A renal biopsy demonstrated focal and segmental necrotising glomerulonephritis with a background chronic parenchymal damage. A diagnosis of MPA was made. She was treated with oral prednisolone and azathioprine, and had an improvement in her renal function (sCr 99 μmol/L), and reduction in MPO- ANCA titer (11 IU/ml).

### Case 5

A 63 year old man was diagnosed with seropositive RA affecting his knees and shoulders and was treated with sulphasalazine. Six years after the diagnosis of RA, he presented with haematuria, proteinuria (2.16 g/24 hours) and decline in renal function (SCr rising from 73 to 148 μmol/L). He had an acute phase response (CRP 70 mg/L, ESR 65 mm/h) and was MPO-ANCA positive (100 IU/ml, normal range 0–6). The RhF was positive (75.5 IU/ml, normal range 0–20). A kidney biopsy demonstrated focal necrotising vasculitic lesions. A diagnosis of MPA was made and he was treated with oral prednisolone and mycophenolate mofetil (MMF) with improvement of his renal function (sCr 106 μmol/L), proteinuria (0.2 g/24 hours) and a reduction in MPO- ANCA titre (10.5 IU/ml).

### Case 6

A 60 year old woman was diagnosed with seropositive RA, after developing symmetrical polyarthritis of the metacarpophalangeal joints and wrists. She was treated with hydroxychloroquine (HCQ), MTX and leflunomide. Disease control was poor, but the patient refused biologic therapy. Seven years later, she presented with haematuria, proteinuria (1.6 g/24 hours) and decline in renal function (SCr rising from 90 to 700 μmol/L) and started hemodialysis. She had an acute phase response (CRP 91 mg/L, ESR 27 mm/h). The RhF was negative. She was ANCA negative, but a renal biopsy demonstrated pauci-immune focal necrotizing glomerulonephritis with moderate chronic damage. A clinical diagnosis of MPA was made. She was treated with oral cyclophosphamide and prednisolone, but did not recover independent renal function. One year later, she died following an episode of a severe sepsis.

Overall, in our six patients there was a median delay of 10.5 years (range 4–43 years) between RA and AAV diagnosis and in all cases the diagnosis of RA preceded the AAV. All had significant renal involvement with AAV, displaying a typical focal and segmental glomerulonephritis, and a clinical diagnosis of MPA predominated. Half were RhF positive at the time of AAV diagnosis, and there was a predominance of MPO-ANCA positivity, as is frequently found in overlap syndromes involving AAV.

## Discussion

We described six patients with rheumatoid arthritis who subsequently developed AAV, mostly with MPA. In addition, we performed a literature search using the terms ANCA, vasculitis and rheumatoid arthritis and found a total of 29 case reports describing this association between AAV and RA (Table 1). Of these cases, 13 patients had an overlap between RA and GPA, 15 RA and MPA and only 1 with RA and EGPA. In all cases, except two, ANCA vasculitis was diagnosed after RA. Of the 29 cases published, 19 patients presented with renal dysfunction, 15 with MPA and 4 with GPA. Immunological testing demonstrated that 26 patients were RhF positive at presentation, while 7 were c-ANCA or PR3-ANCA positive and, 11 were p-ANCA or MPO-ANCA positive. Seven were ANCA negative. Interestingly, ten patients were also ANA positive, suggesting a broader immune dysfunction.

**Table 1:** **Case reports published describing an overlap between ANCA associated vasculitis and rheumatoid arthritis**

**Year/Cases**	**Diagnoses**	**Age RA**	**Age VAS**	**Vasculitis symptoms**	**Positive antibodies**	**RA therapy**	**Follow-up**
1974/1 (Pritchard 1976)	RA/GPA	40	59	ENT	RhF, ANA	Gold, tuberculin injections, NSAID	D
**1976/2** (Ohashi et al. 1992)	RA/GPA	45	45	ENT	RhF	HCQ, aspirin	I
	RA/GPA	73	75	ENT, Eyes, Lung	RhF	Gold, NSAID	I
1**1992/1** (Steuer et al. 1995)	RA/GPA	33	38	ENT	RhF	NSAID, Gold	I
**1995/1** (Harper et al. 1997)	RA/MPA	49	62	Lung,Kidney	RhF, ANA, p-ANCA	NSAID, Gold, SFZ	I
**1997/10** (Messiaen et al. 1998)	RA/MPA	31-62	NA	5: kidney only	4: p-ANCA	6: NSAID	4 D
				1: kidney, lung, eye	9: RhF	1: NSAID, Penicillamine HCQ	2 HD
				3: kidney, skin	6: ANA	1: Prednisone, SFZ, Gold	4 I
				1:kidney, bowel, skin		1: HCQ	
						1: HCQ, SFZ	
**1999/1** (Yorioka et al. 1999)	RA/MPA	22	24	Kidney	RhF, ANA, MPO-ANCA	NSAID	I
**2002/2** (Chinoy & McKenna 2002)	RA/GPA	32	32	ENT, Lung, Kidney	p-ANCA	Prednisolone, NSAID, CYC	I
	RA/GPA	*41	26	Lung	p-ANCA		I
**2003/2** (Douglas et al. 2003)	RA/GPA	36	37	ORL, Lung, Kidney	RhF, PR3-ANCA	Prednisolone, MTX, NSAID.	I
	RA/GPA	35	55	Lung	RhF, PR3-ANCA	NSAID, MTX	I
**2005/1** (Goto et al. 2005)	RA/MPA	44	48	ORL, eyes, Kidney	MPO-ANCA, RhF, ANA	MTX, Penicillamine, Gold, prednisolone	I
**2008/1** (Pai & Panda 2008)	RA/GPA	57	60	Lung	RhF, Anti-CCP, PR3-ANCA	HCQ, prednisolone	I
**2012/4** (Szilasi et al. 2012)	RA/GPA	40	70	Lung, Kidney	PR3-ANCA, Anti-CCP,RhF	Prednisolone	I
	RA/MPA	*49	44	Lung, Kidney	MPO-ANCA, RhF	Adalimumab, RTX	I
	RA/MPA	28	34	Lung, Kidney	MPO-ANCA, RhF	Prednisone, SFZ, MTX	I
	RA/EGPA	52	54	Skin	MPO-ANCA, RhF	MTX, Cyclosporine	I
**2012/1** (Vaishnav et al. 2012)	RA/GPA	36	37	Lung	c-ANCA, RhF		D
**2013/1** (Reitblat & Reitblat 2013)	RA/GPA	45	58	Kidney	PR3-ANCA, RhF	MTX, Etanercept	HD
**2014/1** (Ortiz-Sierra et al. 2014)	RA/GPA	65	67	Skin, lung, ENT, eye	RhF, c-ANCA	MTX, Prednisone, etanercept,	I
Our report	5 RA/MPA	24-63	63-69	1: Kidney, lung	3: RhF	1: MTX, prednisone	5 I
	1 RA/GPA			5: Kidney	1: Anti-CCP	1: SFZ	1 HD
					1: PR3-ANCA	1: HCQ, MTX, leflunomide	1D
					3: MPO-ANCA	1: Infliximab, MTX, SFZ	
						1: Etanercept, MTX, SFZ, AZA	
						1: Prednisolone	

RA: Rheumatoid Arthritis, GPA: Granulomatosis with polyangiitis, MPA: Microscopic polyangiitis, EGPA (Churg-Strauss Syndrome), ENT: ear, nose and throat; HCQ: hydroxychloroquine, NSAID: non-steroidal anti-inflammatory drug, CP: cyclophophamide; AZA: Azathioprine, SFZ: sulfasalazine, MTX: methotrexate, D: dead, I: AAV improved, HD: Hemodialysis.

With regards treatment, for RA we found that hydroxychloroquine, methotrexate, prednisolone and anti-TNF-alpha monoclonal antibodies were most commonly used, while for AAV, cyclophosphamide, pulsed methylprednisolone, and oral prednisolone were most commonly used induction therapies followed by azathioprine as remission maintenance therapy.

Possible reasons for the association between systemic ANCA associated vasculitis and RA may be the common genetic predispositions to autoimmunity which involve the HLA region or genes such as PTPN22, reported in series of both RA and AAV (Jagiello et al. 2005; Farago et al. 2009; Johansson et al. 2006; Lee et al. 2005). In addition, Rocatello et al (Menegattia et al. 2009) showed that polymorphisms in uteroglobin (a multifunctional protein with anti-inflammatory properties) and NF-κB2 (a transcription factors that regulates the expression of a wide range of immune response genes) were associated with a genetic predisposition towards developing both vasculitis and RA.

However, another possibility is the use of TNF antagonists which may have predisposed to development of secondary autoimmune disease. A French nationwide survey identified 39 cases of vasculitis induced following use of TNF antagonists (Saint Marcoux & De Bandt 2006), while a larger series of 379 cases of anti-TNF agent-induced autoimmune diseases and identified 118 patients with vasculitis, most of whom only showed cutaneous leukocytoclastic vasculitis, and 11 (9.3 %) were positive for ANCA (Ramos-Casals et al. 2008). In our study, only 2 patients were treated with anti-TNF alpha (infliximab and etanercept) and subsequently developed MPA.

In summary we have reviewed a total of 35 cases of ANCA associated systemic vasculitis associated with RA and suggest that this may be a rare form of an AAV autoimmune overlap, which includes anti-GBM disease (Weber et al. 1992; Kalluri et al. 1997), scleroderma (Derrett-Smith et al. 2013), SLE (Tetikkurt et al. 2012) and membranous glomerulonephritis (Gaber et al. 1993; Tse et al. 1997). Its recognition could lead to timely antibody screening of patients with the relevant clinical scenario and a more rapid initiation of appropriate management.
